# Identification of Reference Genes for Reverse Transcription-Quantitative PCR Analysis of Ginger Under Abiotic Stress and for Postharvest Biology Studies

**DOI:** 10.3389/fpls.2022.893495

**Published:** 2022-06-06

**Authors:** Gang Li, Jiawei Ma, Junliang Yin, Fengling Guo, Keyong Xi, Peihua Yang, Xiaodong Cai, Qie Jia, Lu Li, Yiqing Liu, Yongxing Zhu

**Affiliations:** ^1^College of Horticulture and Gardening, Spice Crops Research Institute, Yangtze University, Jingzhou, China; ^2^Institute of Economic Crops, Hubei Academy of Agricultural Sciences, Wuhan, China; ^3^School of Environmental Science and Engineering, Suzhou University of Science and Technology, Suzhou, China

**Keywords:** *Zingiber officinale* Roscoe, RT-qPCR, reference gene, internal control, RefFinder

## Abstract

Gene expression analysis largely improves our understanding of the molecular basis underpinning various plant biological processes. Stable reference genes play a foundational role during the normalization of gene expression levels. However, until now, there have been few reference genes suitable for ginger reverse transcription-quantitative PCR (RT-qPCR) research. In this study, 29 candidate reference genes with stable expression patterns across multiple ginger tissues and 13 commonly used reference genes were selected to design RT-qPCR primers. After amplification specificity validation, 32 candidates were selected and further evaluated by RT-qPCR using samples from various organs subjected to NaCl, drought, heat, waterlogging, and chilling stress. Four strategies, including delta-CT, BestKeeper, geNorm, and NormFinder, were used to rank the stability of reference genes, and the ranks produced by these four strategies were comprehensively evaluated by RefFinder to determine the final rank. Overall, the top three stability reference genes indicated by RefFinder were *RBP* > *ATPase* > *40S_S3*. Their expression pattern correlation analysis showed that the coefficients among each pair of *RBP*, *ATPase*, and *40S_S3* were larger than 0.96, revealing consistent and stable expression patterns under various treatments. Then, the expression of three *pathogenesis-related* (*PR*) genes and seven *MYB* genes in rhizomes during postharvest storage and subjected to pathogen infection was normalized by *RBP*, *ATPase*, *40S_S3*, *RBP* and *ATPase*, *ATPase* and *40S-S3*, and *RBP* and *40S-S3*. The results showed that *PR* and *MYB* genes were induced by postharvest deterioration and pathogen infection. The correlation coefficients of *RBP*/*ATPase*, *RBP*/*40S_S3*, *ATPase*/*40S_S3*, *RBP* and *ATPase/ATPase* and *40S-S3*, *RBP* and *ATPase/RBP* and *40S-S3*, and *ATPase* and *40S-S3/RBP* and *40S-S3* were 0.99, 0.96, 0.99, 0.99, 1.00, and 1.00, respectively, which confirmed the stability of these three reference genes in postharvest biology studies of ginger. In summary, this study identified appropriate reference genes for RT-qPCR in ginger and facilitated gene expression studies under biotic and abiotic stress conditions.

## Introduction

Gene expression analysis is an important approach for improving our understanding of the molecular basis underpinning various plant biological processes (Zhu et al., 2019a). RT-qPCR is one of the most frequently used methods for studying gene expression patterns because of its higher sensitivity, accuracy, and reproducibility (Yin et al., 2021). However, due to differences in sample collection, total RNA extraction, mRNA reverse transcription, and PCR procedures, many RT-qPCR experiments are difficult to repeat (Bustin et al., 2009; Kozera and Rapacz, 2013). To eliminate the influence of these adverse factors, some stable expression genes, commonly called reference genes, are used as internal controls to normalize the expression of target genes, which is considered one of the most efficient approaches to correct biases in RT-qPCR studies (Mughal et al., 2018). Reference genes are applied to normalize or quantify gene expression in RT-qPCR analyses. Their expressions are generally expected to remain stable under various experimental treatments, during different growth stages, and in different organs. Therefore, they can be used to correct non-specific variations, such as differences in the quality of extracted RNA and the amount of extracted RNA, the efficiency of cDNA synthesis, and the overall transcriptional activity of the analyzed samples (Kumar, 2014).

Ginger (*Zingiber officinale* Roscoe) contains many bioactive constituents and has been widely used in food, as spices, as flavoring agents, and as medicine (Cheng et al., 2021). Therefore, it has been cultivated in many tropical and subtropical countries, where it has become one of the most economically significant vegetables in the Zingiberaceae family (Yin et al., 2020a). Due to ginger’s economic importance, many studies have been conducted to determine the expression patterns of genes associated with growth and development, abiotic and biotic stress responses, and the quality and quantity of ginger (Peng et al., 2022). Genes, including *TUB2* and *ACT11*, have been used as the internal reference genes in RT-qPCR analysis (Li et al., 2020). Based on previous reports, Lv et al. (2020) selected 14 candidate reference genes and identified *28S* and *COX* as the most stable reference genes in ginger. These 14 candidate reference genes were orthologs of the most commonly used reference genes in other plants, such as a guar (Jaiswal et al., 2019). A suitable reference gene must be expressed steadily in different tissues across all developmental stages and under a wide variety of experimental conditions (Mughal et al., 2018; Yin et al., 2021). However, several reports demonstrate that these commonly used reference genes can lead to inadequate normalization of genes. They may not be expressed constitutively and stably in different tissues or under different conditions in all plant species (Hong et al., 2008; Reddy et al., 2016). Therefore, there is an essential need for system identification and validation of alternative reference genes in ginger to accurately normalize gene expression research. Furthermore, reference genes suitable for gene expression analysis of ginger during postharvest storage are still lacking, which largely constraints research on ginger postharvest biology.

Because of its high resolution, sensitivity, and vast dataset, high-throughput deep RNA-seq has been widely used to decipher molecular biological changes and regulations in plants exposed to a variety of treatments in various tissues (Zhu et al., 2019b; Yin et al., 2021). In recent years, sequence information and the corresponding expression profiling produced by RNA-seq have been employed to identify new reference genes in several plant species, such as grape (González-Agüero et al., 2013), alligator weed (Yin et al., 2021), soybean (Yim et al., 2015), and *Lycoris aurea* (Ma et al., 2016). Most recently, we reported a high-quality, chromosome-scale reference genome of the ginger cultivar “Zhugen,” which laid the foundation for identifying appropriate RT-qPCR reference genes and can be used as an excellent resource for further research on ginger gene expression research (Li et al., 2021).

In this study, we used comprehensive expression profiling results from large-scale RNA-seq data to identify the most stably expressed genes from a variety of ginger tissues subjected to different treatments. Meanwhile, the expression levels of the selected genes under various treatments were further determined using RT-qPCR. Then, the expression stability of selected genes was first ranked by the four algorithms (geNorm, delta-CT, BestKeeper, and NormFinder) and finally ranked by RefFinder by comprehensively considering the ranks produced by the four algorithms. Moreover, the expression patterns of genes related to the postharvest storage of ginger, such as three *pathogenesis*-*related* (*PR*) genes and seven *MYB* genes, were analyzed as a case study to investigate the application of these newly developed reference genes in postharvest biology studies. This study lays the foundation for further gene expression analyses of ginger in future research.

## Materials and Methods

### Ginger Growth and Treatment

A pot culture experiment was performed in the greenhouse of Yangtze University (Jingzhou, Hubei Province, China, 112.026207°E, 30.361273°N). Three *Zingiber officinale* cultivars “Shandongdajiang,” “Yunnanluopingxiaohuangjiang,” and “Fengtoujiang,” which were collected from the North, Southwest, and Central China regions, were sown in 40 silica sand-filled pots (diameter: 20 cm, height: 25 cm). For greenhouse cultivation, the temperature was set to 28/25°C with a photoperiod of 14 h light/10 h dark. After 90 days of planting, healthy seedlings of uniform size (approximately 50 cm in height) were divided into five groups and subsequently subjected to different treatments: (a) water (control), (b) NaCl (2%, w/v), (c) drought (40% FC, field capacity), (d) waterlogging (the pots were submerged 1∼2 cm under the water surface), (e) chilling (10°C), and (f) heat stress (ginger seedlings were treated with 40°C/32°C day/night temperature in a growth chamber) (Yin et al., 2020b). For each treatment, at least 30 seedlings were used per test. After 5 days of treatment, the roots, rhizomes, and leaves were collected and immediately frozen in liquid nitrogen, followed by storage at a temperature of −80°C until further use.

### RNA Isolation and cDNA Synthesis

Total RNA was isolated from samples using the TRIzol reagent (Invitrogen, Carlsbad, CA, United States). The concentration and purity of RNA were evaluated using the NanoDrop Spectrophotometer (NanoDrop Technologies, Wilmington, DE, United States), and their integrity and quality were also analyzed by performing 1.5% (w/v) agarose gel electrophoresis. The cDNA was synthesized using the RevertAid First Strand cDNA Synthesis Kit with oligo-dT and 1 μg of total RNA (Thermo Scientific, Waltham, MA, United States) in a PTC-200 Thermal Cycler (MJ Research Corp., Ltd., Waltham, MA, United States) (Zhu et al., 2020).

### Selection Candidate Reference Genes

In our previous study, Illumina RNA-seq data (accession number PRJNA592215) of four samples collected from the red stem inner portion, green stem, young yellow rhizome, and red stem surface section with three biological replications were produced and used to profile the expression patterns of ginger genes. The Fragments per Kilobase per Million Reads (FPKM), mean values (MV), and standard deviation (SD) of the individual genes were calculated according to 12 data sets. In order to select the considerably high and stable expression candidates, genes with MV values greater than 50 were selected. Then, according to the SD, these selected genes were ranked from the least to the highest SD. Furthermore, based on the functional annotations, 29 candidates with known functions were selected from the top 500 genes. The gene sequences of 13 commonly used reference genes, including *PP2A2* (AT1G10430), *TUBA4* (AT1G50010), *LOS1* (AT1G56070), *UBC35* (AT1G78870), *SAM-Mtase* (AT2G32170), *ACT1* (AT2G37620), *ACT2* (AT3G18780), *TPL40B* (AT3G52590), *UBC10* (AT4G05320), *UBC9* (AT4G27960), *TUBB8* (AT5G23860), *Clathrin* (AT5G46630), and *EF1*α (AT5G60390), were collected and used as a query to perform BLASTn against ginger transcripts with *E*-value < 1e-10 (Vandesompele et al., 2002; Jain et al., 2006; Hong et al., 2008; Kozera and Rapacz, 2013). The best hits were manually extracted, and their expression profiles were also analyzed. The heatmap was illustrated using the R package “ggplot” with the command “pheatmap [log2 (data), color = col, cluster_cols = FALSE, cluster_rows = T),” and box plots were produced using the R function with the command “boxplot (log(data), col = ‘green,’ pars = list (pch = NA, ylab = ‘Average log_2_(FPKM),’ xlab = ‘Gene’)]” (Yin et al., 2021). To further evaluate the expression stability of the 29 selected genes, dot plots were drawn according to the reported method by Yin et al. (2021). Specifically, indicators including coefficient of maximum fold change (MFC, maximum FPKM value/minimum FPKM value) and variation (CV, SD of FPKM/average of FPKM) were calculated and used to produce the dot plot.

### Designing and Specificity Confirmation of Reverse Transcription-Quantitative PCR Primers

Primer Premier 5.0 (PREMIER Biosoft International, Palo Alto, CA, United States) was used to design the gene-specific primers, and exon–exon fragments were specifically targeted while designing the RT-qPCR primers to avoid any possible mismatches and amplification of genomic DNA (Gou et al., 2020). To validate the specificity of primers, a reaction mixture with a total volume of 20 μL was used to perform PCR amplification in an MJ Research PTC-200 thermal cycler. The 20 μL reaction mixture contains 10 μL 2 × Taq Master Mix (Vazyme, Nanjing, China), 3–5 μL DNA template (400 mg L^–1^), 1 μL each of forward and reverse primers (synthesized by GenScript, Shanghai, China), and 5 μL ddH_2_O. The reaction program was set as follows: (1) denaturation at 94°C for 5 min; (2) then denaturation at 94°C for 30 s, annealing at 58°C for 30 s, and extensions at 72°C for 30 s, repeated for 35 cycles; (3) following a final extension at 72°C for 10 min (Yin et al., 2018). Then, 8 μL PCR amplification products were verified by electrophoresis on a 1.5% agarose gel and visualized using the Gel Doc XR system (Bio-Rad, Hercules, CA, United States).

### Reverse Transcription-Quantitative PCR and Data Analysis

RNA samples from ginger cv. “Fengtoujiang” were used to evaluate reference gene stability. RT-qPCR was conducted using SYBR Green Master Mix (Vazyme, Nanjing, China) with the cDNA as a template on a CFX 96 Real-Time PCR system (Bio-Rad, Hercules, CA, United States). The volume of the reaction mixture was 20 μL, which contained 0.4 μL of each of the forward and reverse primers (10 μM), 2.0 μL of diluted cDNA, 10 μL of SYBR Green I Master, and the addition of PCR-grade water up to 20 μL. The RT-qPCR reaction program was set as follows: (1) denaturation at 95°C for 30 s; (2) then denaturation at 95°C for 10 s, annealing at 60°C for 30 s, repeated for 40 cycles; and (3) a melting curve protocol (65–95°C with fluorescence measured every 0.5°C). Three biological replications were adopted for each sample. For RT-qPCR results, RefFinder^1^ was used to evaluate the stability of reference genes under various stresses (NaCl, drought, waterlogging, chilling, and heat) in ginger. Then, samples from cultivars “Shandongdajiang” and “Yunnanluopingxiaohuangjiang” were used to evaluate the stability of the top four stable reference genes. The pairs.panels function in the R package “psych” was used to perform the correlation analysis of the RT-qPCR results of the top four stable reference genes using three ginger cultivars. The standard curve was generated using a set of 10-fold dilutions of cDNA in a RT-qPCR assay according to the equation E = [10^–(1/slope)^ − 1] × 100%. Then, based on the slope of the standard curve, the regression coefficient (*R*^2^) and the RT-qPCR efficiency (E) of primers were calculated (Yin et al., 2021). While preparing and performing RT-qPCR, the Minimum Information for Publication of Quantitative Real-Time PCR Experiments (MIQE) guidelines were followed (Bustin et al., 2009).

### Validation of Candidate References

In order to verify the stability of the top three stable reference genes, two sets of samples were prepared. Uniform sized-young ginger rhizomes (cv. “Shandongdajiang” and “Yunnanluopingxiaohuangjiang”) with no visible injuries or vascular discolorations were selected for further analysis. For postharvest dehydration stress treatment, about 50 ginger rhizomes (cv. “Shandongdajiang”) were held in a growth chamber with 22 ± 2°C and 65% relative humidity conditions. Ginger rhizome samples were collected after 0, 7, 14, 21, and 28 days of postharvest dehydration stress.

*Fusarium solani* strain was isolated from the surface of naturally decaying ginger rhizomes (cv. “Yunnanluopingxiaohuangjiang”) and was cultured [potato dextrose agar (PDA)] at 28°C. A spore suspension (1 × 10^8^ spores mL^–1^) was prepared by washing 5-day-old sporulating cultures with sterile distilled water. For *F*. *solani* infection treatment, the different samples were treated as follows: ginger rhizomes were wounded with a sterilized borer (1.0 cm deep × 0.2 cm wide) at three points around the equator of each rhizome. Each wound was treated with 50 μL of spore suspension. The control rhizomes were wounded and treated with 50 μl sterile water. All treated rhizomes were incubated separately in a plastic box covered with preservative film at 28°C and 90 ± 5% relative humidity for 7 days. Rhizome tissues with a depth of 1.5 cm were collected at the cross area of healthy tissues and disease lesions using a sterilized cork borer. A sample annulus of 1.5–2.0 cm around the wound was collected after 7 days of treatment. All samples were frozen in liquid nitrogen immediately and stored at −80°C. The relative expression of investigated genes was calculated with the 2^–ΔΔCq^ method (Pfaffl, 2001). For the quantification of double reference genes, ΔΔCq was calculated as [((Cqgene – Cqreference1)experiment – (Cqgene – Cqreference1)control)/2 + ((Cqgene – Cqreference2)experiment – (Cqgene – Cqreference2)control)/2 (Gou et al., 2020)].

## Results

### Selection of Candidate Reference Genes

**FIGURE 1:**
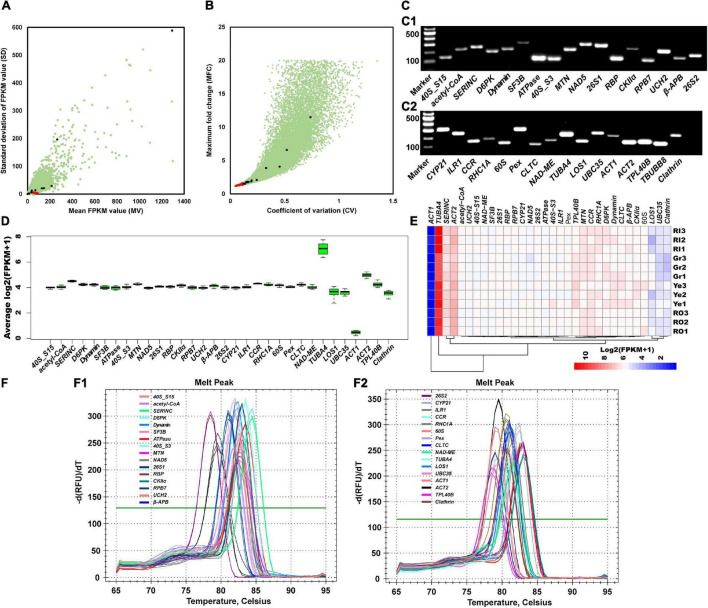
Selection of candidate reference genes. **(A)** SD (standard deviation of FPKM value), MV (mean value of FPKM) values, and **(B)** MFC (maximum fold change) and CV (coefficient of variation) values are used to indicate the expression stability of 32 selected candidate reference genes. Red dots represent the 29 selected stable expression genes, black dots represent the 13 commonly used reference genes, and green dots represent the other genes. **(C)** Primers’ specificity is confirmed by resolving PCR products on an agarose gel. Marker, Marker I. The other lanes belong to the PCR products of candidate reference genes. **(D)** Expression stability overview of 32 selected candidate genes. Box and whisker plot graphs show log_2_(FPKM + 1) values of each selected gene under different treatments in multiple tissues. Black boxes and lines represent the 25th and 75th percentiles and medians, respectively. Whisker caps represent the maximum and minimum values. **(E)** Gene expression levels under different conditions. RI, Gr, Ye, and RO represent ginger samples collected from the inner red stem part, the green stem, the young yellow rhizome, and the red stem surface part. **(F)** Melting peak and melting curve of 32 primers of corresponding candidates to confirm the specificity.

To select suitable candidate reference genes, a set of RNA-seq data was used to determine the expression levels of ginger genes. Gene expression levels were normalized to FPKM values. Then, the FPKM values were used to calculate the mean FPKM (MV) and standard deviation of the FPKM (SD) values. Genes were then ranked from the smallest to the largest according to SD, and 29 genes with MV greater than 50 were selected (Figure 1A and Supplementary Table 1). To estimate their expression stability, the maximum fold change of FPKM (MFC) and coefficient of variation (CV; SD of FPKM/average of FPKM) values were calculated. As shown in Supplementary Table 1, the MFC and CV values of these 29 selected candidates were less than 1.5 and 0.1, respectively (Figure 1B and Supplementary Table 2). Accordingly, the MV, SD, MFC, and CV values revealed that 29 candidate genes were suitable. Meanwhile, commonly used reference genes in *Arabidopsis* were used to perform BLAST to search for homolog genes in ginger, resulting in the identification of another 13 candidate reference genes (Figure 1A and Supplementary Table 3).

Then, 42 pairs of primers with a fragment size of PCR products between 100 and 301 bp were designed according to the sequences of the candidate reference genes (Supplementary Table 4). Their specificity was further checked by PCR amplification and 1.5% (w/v) agarose gel electrophoresis (Figure 1C). As a result, 10 pairs were filtered out due to low specificity (Figure 1C), and 32 pairs of primers (*40S_S15*, *acetyl-CoA*, *SERINC*, *D6PK*, *Dynamin*, *SF3B*, *ATPase*, *40S_S3*, *MTN*, *NAD5*, *26S1*, *RBP*, *CKII*α, *RPB7*, *UCH2*, β*-APB*, *26S2*, *CYP21*, *ILR1*, *CCR*, *RHC1A*, *60S*, *Pex*, *CLTC*, *NAD-ME*, *TUBA4*, *LOS1*, *UBC35*, *ACT1*, *ACT2*, *TPL40B*, and *Clathrin*) were retained for further stability validation. RNA-seq profiling data were further analyzed to illustrate the gene’s expression stability. As shown in the boxplot (Figure 1D) and heatmap (Figure 1E), the expression levels of these 32 candidate genes were fairly stable and consistent under different treatments and among different organs. Candidates that were homologous to commonly used reference genes, such as *TUBA4*, *UBC35*, *ACT1*, and *ACT2*, were less stable than candidates selected based on MV and SD values (Figure 1D). Melting curve analysis showed that a single peak was observed in each pair of primers (Figure 1F). In summary, these results demonstrate that the selected candidate reference genes are stable and that the primers are specific for amplification.

### Identification of Stable Reference Genes

**FIGURE 2:**
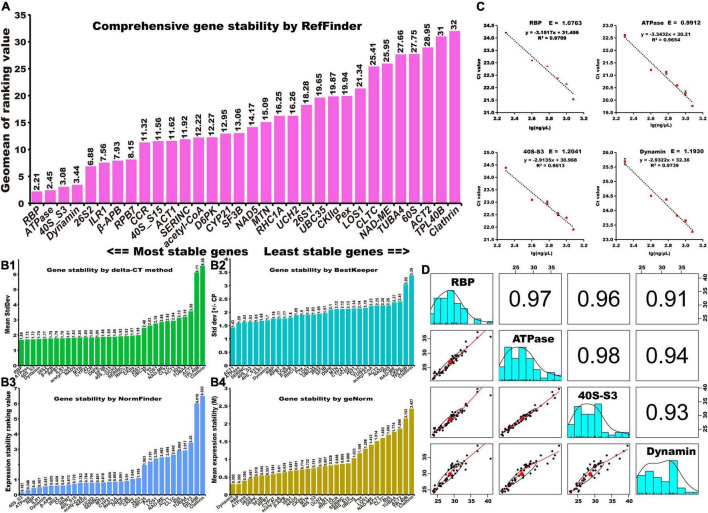
Identification of stable reference genes. Stability rankings of candidate reference genes across samples using five calculation methods: **(A)** RefFinder, **(B1)** delta-CT, **(B2)** BestKeeper, **(B3)** NormFinder, and **(B4)** geNorm. **(C)** The PCR regression coefficient, *R*^2^, and PCR amplification efficiency, E, of *RBP*, *ATPase*, *40S-S3*, and *Dynamin*. **(D)** The stability of *RBP*, *ATPase*, and *40S-S3* was pairwise compared by surveying their expression levels in multiple tissues of three ginger cultivars under five kinds of stress treatments.

Reference genes should be stably expressed in different periods, treatments, and organs (Mughal et al., 2018). To estimate the expression stability of 32 selected reference genes in ginger, their expression was evaluated using RT-qPCR with different cv. “Fengtoujiang” tissues (leaf, rhizomes, and roots) subjected to different treatments (drought, heat, NaCl, waterlogging, and chilling stress) (Supplementary Table 5). Five software tools, namely RefFinder, BestKeeper, NormFinder, geNorm, and delta-CT, were used to identify suitable reference genes. The expression stability of the selected genes was first ranked by the four algorithms of delta-CT, BestKeeper, NormFinder and geNorm (Figures 2B1–B4). As a comprehensive tool, RefFinder can be used to produce the final overall ranking of candidate references according to the geometric mean of the weights of each candidate, calculated by the delta-CT, BestKeeper, geNorm, and NormFinder methods. The top four stability reference genes given by RefFinder were *RBP* > *ATPase* > *40S_S3* > *Dynamin* (Figure 2A).

Then, the PCR efficiencies of *RBP*, *ATPase*, *40S_S3*, and *Dynamin* were calculated. As can be seen in Figure 2C, the amplification efficiency (E) of each primer pair varied between 99.1% for *ATPase* and 120.4% for *40S_S3*. The regression coefficient (*R*^2^) ranged from 0.961 (*40S_S3*) to 0.974 (*Dynamin*). To further validate the stability of *RBP*, *ATPase*, *40S_S3*, and *Dynamin*, their expression levels in the roots, rhizomes, and leaf tissues of another two ginger cultivars, “Shandongdajiang” and “Yunnanluopingxiaohuangjiang,” under different stress conditions were determined by RT-qPCR (Supplementary Table 6). Then, the Cq values generated from the three cultivars were collected to perform a correlation analysis (Figure 2D). The results showed that, without *Dynamin*, the relative coefficient between each pair of *RBP*, *ATPase*, and *40S_S3* was larger than 0.96, suggesting that *RBP*, *ATPase*, and *40S_S3* are favorable reference genes for RT-qPCR studies for ginger. Primer information for *RBP*, *ATPase*, and *40S_S3* is listed in Table 1.

**TABLE 1:** Detailed primer information for *RBP*, *ATPase*, and *40S_S3*.

Primer_ID	Primer sequences (5′-3′)	Length (bp)	Tm (°C)	Amplicon length (bp)
RBP-F	CCTATGAAGCGTAGAAACACAAG	23	60	123
RBP-R	GGAAGGACAACATCCCAAATC	21		
ATPase-F	CGTGGGAGATGCTGGAG	17	60	119
ATPase-R	GGATGATGCCGACAATGAG	19		
40S_S3-F	GGACGGAGTGTTCTTTGC	18	60	113
40S_S3-R	GGATAATGATCTCGGTTCG	19		

### Application of Reference Genes in the Postharvest Study of Ginger

**FIGURE 3:**
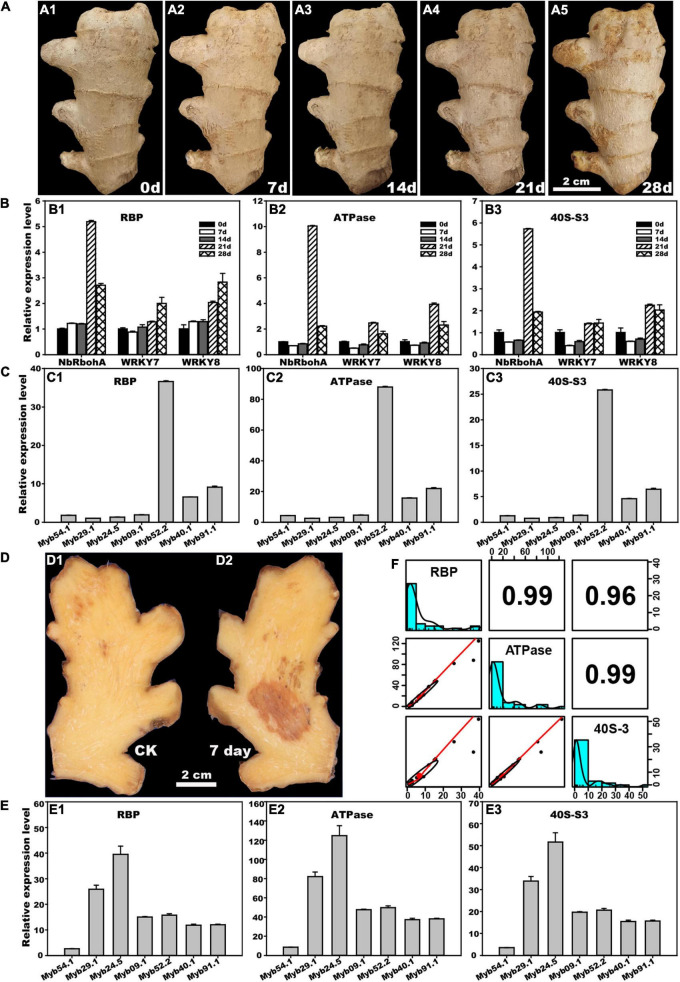
Application of stability reference genes *RBP*, *ATPase*, and *40S-S3* to postharvest ginger. **(A)** Natural deterioration of ginger rhizome during 0 to 28 days postharvest storage. **(B)** Changes in relative expression levels of three *pathogenesis*-*related* genes during rhizome postharvest storage determined by *RBP*, *ATPase*, and *40S-S3*. **(C)** Changes in the relative expression level of seven flavonoid-related *MYB* genes during rhizome 7 days natural postharvest storage determined by *RBP*, *ATPase*, and *40S-S3*. **(D)** Disease lesion of the rhizome after inoculation by *Fusarium solani* for 7 days. **(E)** Changes in the relative expression level of seven flavonoid-related *MYB* genes after rhizome inoculation by *F*. *solani* for 7 days determined by *RBP*, *ATPase*, and *40S-S3*. **(F)** Validation stability by correlation analysis of the relative expression levels of three *pathogenesis*-*related* and seven flavonoid-related genes determined by *RBP*, *ATPase*, and *40S-S3* above.

As shown in Figure 3A, the rhizome gradually deteriorated during the 28 days of postharvest storage. The expression levels of three *pathogenesis-related* (*PR*) genes, *NbRbohA*, *WRKY7*, and *WRKY8*, were determined by *RBP*, *ATPase*, and *40S-S3*, respectively (Figure 3B). Generally, the expression patterns of the three *PR* genes determined by the three reference genes were similar. The expression of *NbRbohA* normalized by *RBP* was slightly increased on days 7 and 14 of storage but significantly increased on days 21 and 28, whereas its expression levels normalized by *ATPase* and *40S-S3* were slightly decreased on days 7 and 14 but significantly increased on days 21 and 28. The expression of *WRKY7* and *WRKY8* normalized by *ATPase* and *40S-S3* showed similar trends. Their expression decreased on days 7 and 14 but increased on days 21 and 28. The expression of *WRKY7* normalized by *RBP* decreased on day 7 but increased after 14 days of treatment. The expression of *WRKY8* normalized by *RBP* increased throughout treatment.

*MYB* genes are functionally associated with lignin synthesis and many other growth processes (Chen et al., 2021). The RT-qPCR analysis revealed that the expression of seven *MYB* genes in postharvest storage samples, and *F*. *solani* inoculation samples showed similar trends when normalized by *RBP*, *ATPase*, and *40S-S3*. In postharvest storage samples, *Myb52*.*2*, *Myb91*.*1*, and *Myb40*.*1* were upregulated after 7 days of storage, with *Myb52*.*2* being the largest upregulated gene. The other four genes, *Myb54*.*1*, *Myb29*.*1*, *Myb24*.*5*, and *Myb09*.*1*, were normally expressed after 7 days of storage in the ginger rhizome (Figure 3C). Meanwhile, the expression of seven *MYB* genes in ginger under *F*. *solani* inoculation rhizome for 7 days was analyzed (Figure 3D), and the results showed that, besides *Myb54*.*1*, the other six genes *Myb29*.*1*, *Myb24*.*5*, *Myb09*.*1*, *Myb52*.*2*, *Myb91*.*1*, and *Myb40*.*1* were upregulated, especially for *Myb24*.*5* and *Myb29*.*1*, which were the top two sharply upregulated *MYB* genes (Figure 3E).

To further validate the stability of *RBP*, *ATPase*, and *40S_S3* during their application to postharvest storage studies, *PR* and *MYB* gene expression levels in ginger rhizomes determined by RT-qPCR were used to perform correlation analysis (Figure 3F). The results showed that the relative coefficients between each pair of *RBP*, *ATPase*, and *40S_S3* were greater than 0.96, which further confirmed that *RBP*, *ATPase*, and *40S_S3* are favorable RT-qPCR reference genes for postharvest biology studies in ginger rhizomes.

**FIGURE 4:**
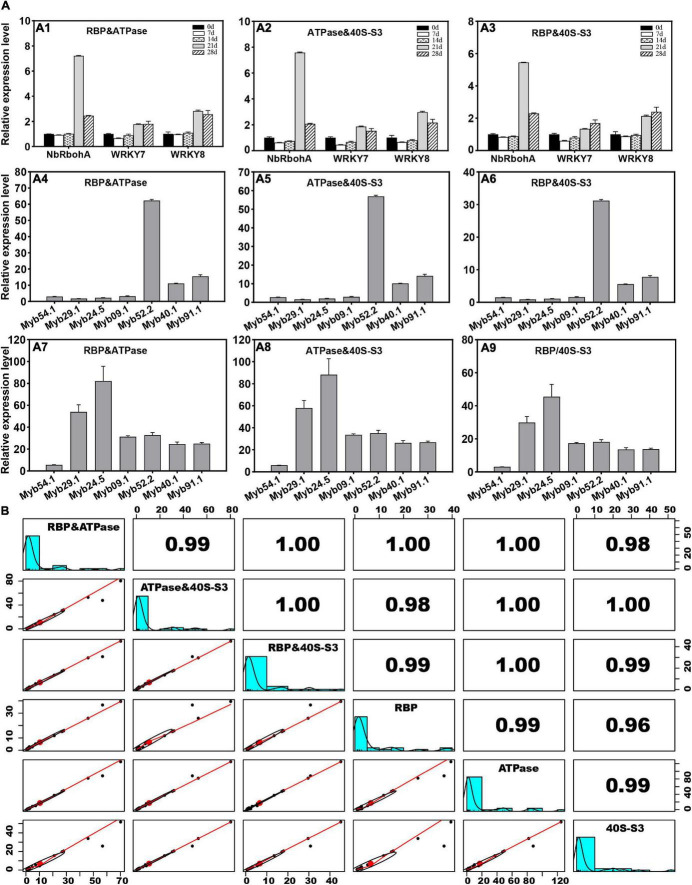
Estimation of the stability of the double reference gene method. **(A)** The expression levels of *PR* and *MYB* genes are determined by reference gene pairs of *RBP* and *ATPase*, *ATPase* and *40S-S3*, or *RBP* and *40S-S3*. **(B)** Correlation analysis of expression levels determined by two and single reference genes.

Furthermore, the expression levels of *PR* and *MYB* genes were further analyzed with double reference genes, including *RBP* and *ATPase*, *ATPase* and *40S-S3*, and *RBP* and *40S-S3*. The results showed that the expression patterns of these genes standardized by double reference genes were consistent with those calculated by a single reference gene (Figures 4A1–A9). The results showed that the relative coefficient between each pair of *RBP* and *ATPase*, *ATPase* and *40S-S3*, and *RBP* and *40S-S3* was greater than 0.96 (Figure 4B). The relative coefficient between *RBP* and *ATPase* and *ATPase* and *40S-S3* was 0.99. The relative coefficient between *RBP* and *ATPase* and *RBP* and *40S-S3* was 1.00. The relative coefficient between *ATPase* and *40S-S3* and *RBP* and *40S-S3* was 1.00.

## Discussion

There are many constraints in the ginger planting process, such as heat, chilling, drought, waterlogging, and salt stress, and the rhizome deteriorates after harvest due to dehydration, pathogen invasion, and senescence (Lv et al., 2020). Quantification of the expression patterns of key genes by RT-qPCR is an important and commonly used approach for deciphering the response mechanism of ginger under different conditions (Fan et al., 2013; Zhu et al., 2019a). Apparently, ideal reference genes are crucial to the reliability and accuracy of the RT-qPCR analysis (Mughal et al., 2018). Until now, evaluation and validation of reference genes have been performed in many plants, but limited information is available on stable reference gene development for ginger. Furthermore, there is still no report about using RNA-seq datasets to facilitate the development of stable expression reference genes in ginger.

### RNA-Seq Data Boosted the Selection of Candidate Stable Reference Genes

In recent years, expression information and assembling sequences produced by RNA-seq have been used to identify novel and stable reference genes for several plant species (Leebens-Mack et al., 2019; Yin et al., 2021). Previously, we produced a set of transcriptome data and determined the expression levels of ginger genes in various tissues. In this study, the expression profiling data were used to develop appropriate internal reference genes for the quantification of genes in ginger, with specific attention to the application of reference genes in studies associated with stress and rhizome postharvest storage.

Suitable reference genes must meet three main criteria, as proposed by Mughal et al. (2018): (1) The genes must be above the basal cell background (moderate to a high level of expression); (2) the genes must exhibit expression in different developmental stages, various physiological states, and multiple tissues; and (3) the gene expression levels show low variation among different tissues and developmental, physiological, and environmental conditions. Accordingly, to select suitable candidate reference genes, the expression levels of ginger genes determined by RNA-seq were normalized to FPKM values, which were then used to calculate the SD (FPKM standard deviation) and MV (FPKM mean value) values. Genes were further ranked from smallest to largest according to the SD value, and 29 candidates with MV values greater than 50 were selected from the top-ranking genes (Figure 1A and Supplementary Table 1). Meanwhile, MFC (FPKM maximum fold change) and CV (coefficient of variation, SD of FPKM/average of FPKM) values were also reported as crucial indicators for estimating the expression stability of candidates. Thus, the MFC and CV values of the candidates were further calculated; the results showed that the MFC and CV of these 29 selected candidates were smaller than 1.5 and 0.1, respectively (Figure 1B and Supplementary Table 2). The SD, MV, MFC, and CV indicators suggested that these 29 selected candidate genes were suitable for subsequent reference primer development.

Interestingly, those commonly used reference genes, such as *Actin*, *Tubulin*, *EF1*α, and *Ubiquitin* (Yin et al., 2021), were not found in the 29 candidates. By querying commonly used reference genes in *Arabidopsis* against the ginger genome, 13 ginger homologs were manually identified and used as candidate references (Supplementary Table 3). According to SD, MV, MFC, and CV values (Figures 1A,B,D,E), the expression patterns of these 13 candidates varied across different tissues and environmental conditions, implying that the candidates lacked stability and were not suitable for use as references. Similar to these results, previous studies also reported that these commonly used reference genes were not always stably and constitutively expressed in different tissues, treatments, and species (Hong et al., 2008; Reddy et al., 2016). For example, Reddy et al. (2016) reported that the newly developed reference genes performed significantly better than the traditionally used reference genes, such as *Actin* and *EF1*α. Hong et al. (2008) found that the most commonly used reference gene, *Actin*, was inappropriate for use as a reference gene in *Brachypodium distachyon*, as some variations of the transcript levels were detected in different plant tissues and under different growth conditions. However, to compare the stability of newly selected candidates with that of traditionally used reference genes, we retained the 13 candidates and conducted further primer design and stability assessments.

### *RBP*, *ATPase*, and *40S_S3* Were Validated to Be the Most Stable Reference Genes

Reference genes should be stably expressed in different periods, treatments, and tissues (Yin et al., 2021). To validate the expression stability and screen the best ones for gene expression normalization in ginger, we then evaluated the expression levels of 32 candidate reference genes in different samples using RT-qPCR (Supplementary Table 5). To improve the reliability of the assessment of reference genes, we used four algorithms (BestKeeper, NormFinder, geNorm, and delta-CT) and a combination of the four statistical methods called RefFinder in the present study to identify stable reference genes. However, the ranking orders of the 32 candidate reference genes determined by the five algorithms showed some differences, mainly because of the different statistical strategies. Ranking order differences have also been found in several other studies. For example, Duan et al. (2017) found that, due to differences among the algorithms, the most unstable genes identified by the four algorithms were mostly the same, whereas the ranking order of the most stable genes was different. Similarly, in this study, *Clathrin* and *TPL40B* were indicated to be the most unstable genes in the four algorithms (BestKeeper, NormFinder, geNorm, and delta-CT), while the order of stable genes was slightly different. RefFinder is a comprehensive tool that can be used to produce a final overall ranking of candidate reference genes according to the geometric mean of the weights of every gene generated by the NormFinder, BestKeeper, geNorm, and delta-CT programs (Xie et al., 2012). Thus, RefFinder was used to produce the final ranking order, and the final top four stability reference genes ranked by RefFinder were *RBP* > *ATPase* > *40S_S3* > *Dynamin* (Figure 2A).

Similar to previous studies (Hong et al., 2008; Reddy et al., 2016), in this study, we also found that our newly developed reference genes performed better than the traditionally used reference genes. Comprehensive ranking using RefFinder showed that *RBP*, *ATPase*, *40S_S3*, and *Dynamin* were ranked as the top four stable genes, while traditional references, such as *ACT2*, *TUB4*, *UBC35*, and *LOS1*, were ranked as the most unstable genes in the sample sets. Traditional reference genes are widely used in plant molecular biology studies, but the stability of these genes needs to be verified in more plant species, in different tissues, and under various conditions. Based on this study and many other studies, RNA-seq data should be used to develop novel reference genes. Moreover, at least two reference genes are highly recommended because only one may lead to relatively large errors (Kozera and Rapacz, 2013; Mughal et al., 2018).

PCR efficiency analysis of *RBP*, *ATPase*, *40S_S3*, and *Dynamin* confirmed that the corresponding primers had high amplification efficiency and were suitable for RT-qPCR analysis (Figure 2C). Furthermore, the stability of *RBP*, *ATPase*, *40S_S3*, and *Dynamin* was confirmed using two more ginger cultivars subjected to different stress conditions (Supplementary Table 6). Correlation analysis of Cq values obtained from three cultivars showed that, besides *Dynamin*, the relative coefficient between each pair of *RBP*, *ATPase*, and *40S_S3* was larger than 0.96. These results revealed that the relative expression levels determined by *RBP*, *ATPase*, and *40S_S3* are fairly similar, and they are the optimal reference genes for ginger gene expression analysis in different cultivars, tissues, and experimental conditions.

### *RBP*, *ATPase*, and *40S_S3* Performed Well in Postharvest Ginger Genes’ Quantification

The rhizome is an economically crucial part of ginger and usually goes through one or more years of storage. During postharvest storage, microorganisms are important causal agents of spoilage. *MYB* transcription factors (TFs) participate in many developmental and physiological processes, including modulating lignin biosynthesis and plant resistance against pathogens (Zhang et al., 2019; Chen et al., 2021). To further test the stability and possible usage of selected reference genes in the postharvest biology study of ginger rhizomes, *RBP*, *ATPase*, and *40S-S3* were used to normalize the expression patterns of three *pathogenesis-related* (*PR*) genes (including two PTI-related genes, *WRKY7* and *WRKY8*, and one reactive oxygen-related gene, *NbRbohA*) and seven *MYBs* (Yin et al., 2020c; Chen et al., 2021). Ginger rhizomes gradually deteriorated during 28 days of postharvest storage, which may be due to microbial infection. Gene expression analysis of three *PR* genes, *NbRbohA*, *WRKY7*, and *WRKY8*, showed similar expression patterns at different time points during storage when standardized by three reference genes (*RBP*, *ATPase*, and *40S-S3*). Similarly, seven *MYB* genes that were potentially functionally associated with lignin synthesis were upregulated in *F. solani*-inoculated rhizomes, regardless of which reference genes (*RBP*, *ATPase*, or *40S-S3*) were used (Figure 3E). Similarly, the stability of *RBP*, *ATPase*, and *40S_S3* was tested using postharvest storage samples, and the results showed that seven *MYB* genes had similar expression trends, regardless of which reference genes (*RBP*, *ATPase*, and *40S-S3*) were used. For further validation, correlation analysis was performed using the expression data of three *PR* and seven *MYB* genes determined by *RBP*, *ATPase*, and *40S_S3* (Figure 3F). The pairwise coefficients of *RBP* and *ATPase*, *RBP* and *40S_S3*, and *ATPase* and *40S_S3* were 0.99, 0.96, and 0.99, respectively, which confirmed the stability of the three selected reference genes in the postharvest biology study of ginger rhizomes. Moreover, as recommended by Kozera and Rapacz (2013), at least two reference genes should be used during gene quantification studies because using one could result in significant biases. For example, Derveaux et al. (2010) indicated that three-fold bias was detected in 25% of the analyzed results or even six-fold bias in 10% of the quantification results. Thus, in this study, the expression patterns of 10 selected genes were further analyzed with double reference genes, including *RBP* and *ATPase*, *ATPase* and *40S-S3*, and *RBP* and *40S-S3*. As shown in Figure 4A, the expression patterns determined by double reference genes showed a similar tendency as those determined by single reference genes, i.e., *RBP*, *ATPase*, and *40S-S3* (Figures 3B,C,E). Furthermore, the correlation analysis of expression levels determined by reference gene pairs of *RBP* and *ATPase*, *ATPase* and *40S-S3*, or *RBP* and *40S-S3* reached 0.99 (Figure 4B). Accordingly, based on our results, each pair of *RBP*, *ATPase*, and *40S-S3* is suitable for double reference genes for the RT-qPCR study of ginger, especially for the determination of the expression pattern of genes functionally associated with ginger postharvest biological responses.

## Conclusion

In this study, three stably expressing genes, *RBP*, *ATPase*, and *40S_S3*, were identified as suitable for the accurate normalization of target gene expression in ginger across all experimental subsets. *RBP*, *ATPase*, and *40S_S3* were also recommended for application to gene expression analyses in postharvest biology studies of ginger rhizomes. Our results support the importance of using high-throughput transcriptomic data to select new reference genes in RT-qPCR experiments, as traditionally used reference genes did not show stable enough expressions in ginger. Our identification of optimal reference genes could make an important contribution to qPCR-based gene expression analysis for ginger functional genomics.

## Data Availability Statement

The datasets presented in this study can be found in online repositories. The names of the repository/repositories and accession number(s) can be found in the article/Supplementary Material.

## Author Contributions

GL, JY, and YZ designed the experiments. GL, JM, KX, and PY conducted the experiments and analyzed the data with supervision by FG, XC, QJ, and LL. JY wrote the manuscript. All authors read and approved the manuscript.

## Conflict of Interest

The authors declare that the research was conducted in the absence of any commercial or financial relationships that could be construed as a potential conflict of interest.

## Publisher’s Note

All claims expressed in this article are solely those of the authors and do not necessarily represent those of their affiliated organizations, or those of the publisher, the editors and the reviewers. Any product that may be evaluated in this article, or claim that may be made by its manufacturer, is not guaranteed or endorsed by the publisher.

## Footnotes

1
http://blooge.cn/RefFinder/

## Supplementary Material

Supplementary Table 1 FPKM candidate.

Supplementary Table 2 Blast candidate.

Supplementary Table 3 FPKM annotation candidate.

Supplementary Table 4 Candidate primer information.

Supplementary Table 5 Fengtoujiang Cq values.

Supplementary Table 6 Other cultivars Cq values.

Supplementary Table 7 *PR* and *MYB* primer information.

Supplementary Table 8 *PR* and *MYB* Cq values.
